# Rapid Improvement in Visual Selective Attention Related to Action Video Gaming Experience

**DOI:** 10.3389/fnhum.2018.00047

**Published:** 2018-02-13

**Authors:** Nan Qiu, Weiyi Ma, Xin Fan, Youjin Zhang, Yi Li, Yuening Yan, Zhongliang Zhou, Fali Li, Diankun Gong, Dezhong Yao

**Affiliations:** ^1^The Clinical Hospital of Chengdu Brain Science Institute, MOE Key Lab for Neuroinformation, University of Electronic Science and Technology of China, Chengdu, China; ^2^School of Life Science and Technology, Center for Information in Medicine, University of Electronic Science and Technology of China, Chengdu, China; ^3^School of Human Environmental Sciences, University of Arkansas, Fayetteville, AR, United States

**Keywords:** action video game, neural plasticity, visual selection attention, electrophysiological measures, P3 amplitude

## Abstract

A central issue in cognitive science is understanding how learning induces cognitive and neural plasticity, which helps illuminate the biological basis of learning. Research in the past few decades showed that action video gaming (AVG) offered new, important perspectives on learning-related cognitive and neural plasticity. However, it is still unclear whether cognitive and neural plasticity is observable after a *brief* AVG session. Using behavioral and electrophysiological measures, this study examined the plasticity of visual selective attention (VSA) associated with a 1 h AVG session. Both AVG experts and non-experts participated in this study. Their VSA was assessed prior to and after the AVG session. Within-group comparisons on the participants' performance before and after the AVG session showed improvements in response time in both groups and modulations of electrophysiological measures in the non-experts. Furthermore, between-group comparisons showed that the experts had superior VSA, relative to the non-experts, prior to the AVG session. These findings suggested an association between the plasticity of VSA and AVG. Most importantly, this study showed that the plasticity of VSA was observable after even a 1 h AVG session.

## Introduction

Learning is essential for cognitive development. The mechanism of learning and the acquisition of abilities is therefore a central issue in cognitive science. Research over the past few decades has shown that the brain changes physically, functionally, and chemically as one acquires or improves an ability (Hultsch et al., 1999; Salthouse, 2006; Ackerman et al., 2010; Lövdén et al., 2010; Shors et al., 2012; Gong et al., 2013; Thomas and Baker, 2013; Connors et al., 2014; Yang et al., 2014). Recent progress in cognitive science demonstrates that action video gaming (AVG) is associated with cognitive and neural plasticity, thus offering important, new insights into the neural basis of learning (Powers et al., 2013; Cardoso-Leite and Bavelier, 2014; Gong et al., 2016). The AVG-related plasticity may be due to the fact that AVG typically requires one to process complex sequences of events and respond to various stimulus accurately and rapidly (Latham et al., 2013; Gong et al., 2015). AVG is therefore a cognitively demanding task, for which visual selective attention (VSA) is critical (Green and Bavelier, 2015), as VSA guides attention toward the task-relevant information and away from the irrelevant or distracting information (Serences et al., 2004; Bavelier et al., 2012). This study examines the effects of brief and long-term AVG on measures of VSA.

While a few research groups have failed to observe positive results from action video game training on cognitive abilities (Owen et al., 2010; Boot et al., 2013; Kundu et al., 2013), meta-analyses indicate a clearly significant overall effect in the medium to large range on the association between AVG and cognitive development (Latham et al., 2013; Powers et al., 2013; Toril, 2014; Wang et al., 2016). Using behavioral methods, research found that AVG was related to improvements in VSA. For example, AVG experts scored higher than non-experts on measures of VSA in tasks like flanker compatibility, enumeration, useful field of view, and attentional blink (Bavelier et al., 2012). In addition, interventional research found that AVG training was related to improved performance on the useful field of view (UFOV) and attentional blink tasks (Green and Bavelier, 2003, 2006).

The UFOV is the visual area over which information can be extracted at a brief glance without eye or head movements (Ball et al., 2002). The UFOV assessment has been widely used to study VSA, including processing speed, divided attention, and selective attention in detecting the appearance of target stimulus (Feng et al., 2007; West et al., 2008; Krishnan et al., 2013; Oei and Patterson, 2014). Participants are typically asked to indicate the eccentricity (5, 10, 15°) at which the target stimulus appears. In the UFOV task, the target stimulus appears unpredictably but equally often in one of 24 different peripheral locations (Feng and Spence, 2007; Sungur and Boduroglu, 2012). The 24 locations are arranged into eight evenly spaced radial spokes (Sekuler et al., 2000). Using the center location of a spoke as the reference point, there are three locations on each side of the spoke, which are at the eccentricities of 5, 10, and 15° respectively. Thus, the stimulus display is often described as three concentric circles at the visual angles of 10, 20, 30° respectively (Green and Bavelier, 2003; Sungur and Boduroglu, 2012; Boduroglu and Shah, 2017). Using the UFOV assessment, research found that the participants who received AVG training outperformed those who did not receive such training (Green and Bavelier, 2003; Green et al., 2010).

Thus, AVG experts have superior VSA compared to AVG non-experts (Green and Bavelier, 2003; Bavelier et al., 2012; Bejjanki et al., 2014). This superior VSA may optimize the use of cognitive resources, thus enabling AVG experts to respond to stimulus more accurately and rapidly (Dye et al., 2009). Furthermore, AVG is associated with the improvement of cognitive abilities that are highly relevant to VSA, as research indicated that AVG experts had better perception threshold and processing speed (Schubert et al., 2015), visual sensitivity (Appelbaum et al., 2013), visual short-term memory storage (Colzato et al., 2012; Blacker and Curby, 2013; Blacker et al., 2014), top-down guidance in visual search (Wu and Spence, 2013), spatial distribution of attention (Feng et al., 2007; West et al., 2008), and oculomotor control (West et al., 2013) than AVG non-experts.

Neuroscience research has also examined the effects of AVG on VSA. An electroencephalography (EEG) study found that a 10 h AVG program was associated with modulated P2 and P3 potentials and improved VSA (Wu et al., 2012). As P3 potentials may indicate the amount of employed mental resources (Drollette et al., 2014), P3 is related to the use of attention resources (Donchin and Coles, 1988; Milne et al., 2013). Using steady-state visually evoked potentials, research showed that AVG players were less likely to be distracted by irrelevant information than non-players (Krishnan et al., 2013). A recent fMRI study also showed that AVG experts had enhanced functional integration between the salience network and the central executive network – two critical neural networks for VSA (Gong et al., 2016). These studies used AVG programs ranging from several hours (Feng et al., 2007) to several months (Wu et al., 2012) in duration. Thus, it was still unclear whether cognitive and neural plasticity is observable after a *brief* AVG session. An examination of this issue can improve our knowledge of the amount of AVG experience required for neural plasticity, which is central to any complete theory of neural plasticity.

Using behavioral and EEG measures, this study examined whether VSA plasticity is observable after a 1 h AVG session. Then, we further identified the cognitive functions and electrophysiological markers that are closely related to AVG. This study used EEG because of its high temporal resolution (Carlino et al., 2012), which allowed us to examine temporally sensitive indicators. Both AVG experts and non-experts were asked to complete a 1 h AVG session and their VSA was assessed prior to (the pre-AVG phase) and after (the post-AVG phase) the AVG session. We predicted that the experts would have VSA superior to that of the non-experts prior to the AVG session, and that VSA would be improved after the AVG session. This study analyzed ERPs, the EEG power spectrum energy, and behavioral measures.

## Materials and methods

### Participants

Prior to this study, a survey was given to a large group of individuals who were asked to report their League of Legends (LOL) gaming experience (in years) and their Expertise Ranking that was provided by the LOL game—the AVG program used in this study. Only the individuals who were identified as either experts or non-experts were invited to participate in this study. The participants were 29 males, healthy undergraduate and graduate students from the University of Electronic Science and Technology of China (UESTC). Both AVG experts (*M* = 22.26 ± 0.23 years; *n* = 15) and non-experts (*M* = 23.30 ± 0.15 years; *n* = 14) were recruited. All participants were right-handed as confirmed by the Edinburgh Handedness Questionnaire (Oldfield, 1971). They provided informed consent and the test was approved by the UESTC Ethics Board. To minimize participant bias, the participants were not notified of their group membership or the purpose of this study.

The group membership was defined based on both time- and skill-based criteria. The experts had at least 2 years of AVG experience and were recognized as AVG masters according to the Expertise Ranking (the top 7% players) provided by the LOL—an objective, widely used method for calculating the relative skill levels of LOL players^1^. The non-experts had less than 0.5 years AVG experience and were recognized as amateurs based on their rankings (the lowest 29.92~45.11% players). Unlike past research that recruited non-AVG players who had no AVG experience (Green and Bavelier, 2003; Li et al., 2009; West et al., 2015), this study tested non-experts who had some AVG experience. This selection criterion was used to minimize the influence of novel experience on non-experts' performance.

Furthermore, the recruitment of both AVG experts and non-experts enabled us to address three issues. First, the association between a 1 h AVG experience and VSA plasticity can be illuminated through comparisons of participants' pre-AVG and post-AVG VSA performances. Second, the relationship between long-term AVG experience and VSA can be addressed through between-group comparisons of the participants' pre-AVG performance. Finally, the interaction between long-term and brief AVG experience can be revealed by between-group comparisons of participants' post-AVG performance.

### Stimulus and procedure

#### Stimulus preparation and overall procedure

**Figure 1:**
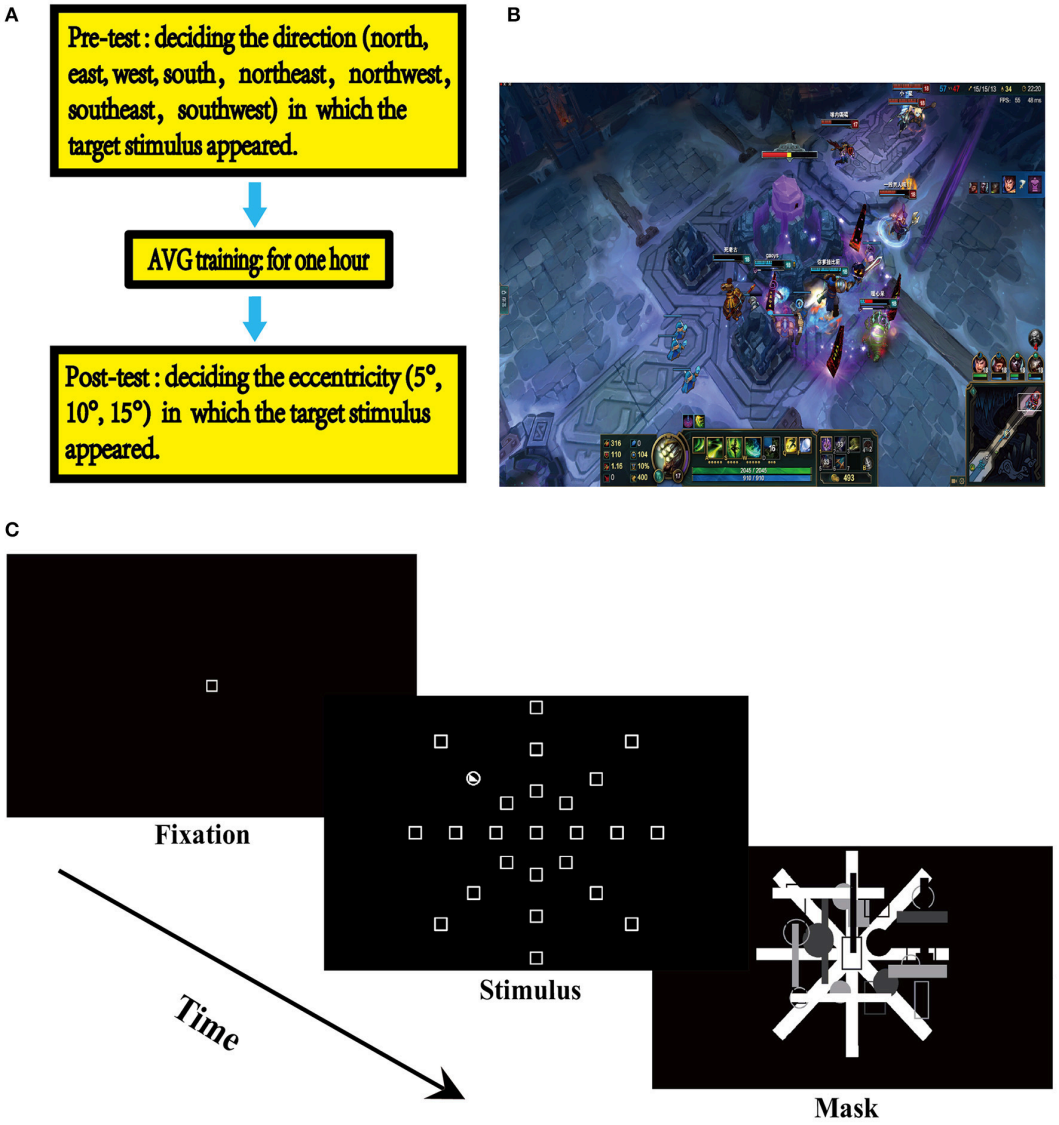
Experimental design. **(A)** Experimental design. **(B)** League of Legends (LOL). **(C)** The useful field ofview (UFOV) task.

An experiment consisted of three sequential phases: pre-testing on the UFOV, a 1 h gaming session, and post-testing on the UFOV (Figure 1A). Two different UFOV assessments were used to minimize the test-retest practice effect. The Microsoft image editor - Paint - was used to create visual stimulus for the UFOV assessments. In the UFOV test, the participants were seated in front of the screen at a distance of approximately 57 cm from the center of the screen (Gross et al., 1972). At this viewing distance, a distance between two locations on the screen of 1 cm corresponds to one degree of visual angle (Sungur and Boduroglu, 2012). The UFOV tasks were presented using E-prime 2.0. Each UFOV assessment consisted of three test blocks, each containing 120 trials. The presentation order of trials was randomized within each block, while the presentation order of test blocks was counterbalanced across participants. The monitor (Model: L2250pwD, 22 inches, Height: 30 cm, Width: 47 cm) was placed in the testing room and was used to display stimuli for the participants.

#### Pre-testing on the UFOV

Prior to the AVG session, the participants completed a UFOV assessment, where they were asked to detect the direction in which a stimulus appeared (north, northeast, northwest, east, west, south, southeast, southwest). Participants were seated in a dimly lit room, sound attenuated, and electrically shielded testing booth. In the UFOV tasks, each trial started with the presentation of a fixation square in the center of the display area which remained for 1,000 ms. Then, the target stimulus was presented and remained for 100 ms. Then, a masking stimulus was presented, which was used to eliminate the interference of the visual afterimage phenomenon. Participants were asked to respond accurately and quickly to the target stimulus by pressing a key or operating a game joystick on a control panel consisting of a joystick and keys. The presentation of the masking stimulus lasted until the participants responded to the target stimulus. In the pre-AVG phase, participants were asked to indicate north, east, west, and south by operating the joystick that is maneuverable in the four directions, or to indicate northeast, northwest, southeast, and southwest by pressing a key assigned for that particular direction (Figure 1C).

#### Game play

The participants then completed a 1 h AVG session where the LOL was used (Figure 1B). The LOL belongs to the Multiplayer Online Battle Arena (MOBA) genre—commonly known as Action Real-time Strategy games (Action-RTS games) (Dale and Green, 2017b), as MOBA games contain elements of both action video games and RTS games. Since both action games and RTS games have been associated with certain cognitive improvements, it is arguable that MOBA games may also be associated cognitive improvements (Glass et al., 2013; Dale and Green, 2017a). This study used Normal 5v5 Matchmaking—a LOL game model referred to as Howling Abyss—where two teams, each consisting of 5 players, competed with each other. The participants completed the AVG session individually in this study; thus, each participant was teamed with four other players assigned by the game, competing against the other team that was also randomly assigned by the game. The feature of Random Pick was activated, which randomly assigned a champion to each player. Howling Abyss is a group fighting mode that requires collaboration. Therefore, players were required to coordinate eye, hand, and attention and to collaborate with the other players on their team.

#### Post-testing on the UFOV

After the AVG session, the participants completed another UFOV assessment, where they were asked to indicate the eccentricity (10, 20, 30°), from which a stimulus appeared, by pressing a key assigned for that particular eccentricity (Figure 1C). Each UFOV assessment lasted approximately 10 min. There were 10 min breaks after both the pre-AVG UFOV assessment and the AVG session. An experimental session lasted approximately 90 min. This experiment is a mixed design, and participants performed the UFOV tasks using the ABBA balance method.

### EEG recording and data analysis

EEG data were collected with an electrode cap of 64 Ag-AgCl electrodes. Electrode position was based on the 10–20 system (Jasper, 1958) and digitized with a sampling rate of 500 Hz (Brain Products GmbH). The impedance for all electrodes was kept below 5 kΩ, and all the data were online filtered with a 0.01–100 Hz bandpass filter. All channels were recorded with frontal vertex (i.e., FCz) as the reference and converted to linked-mastoid reference off-line. AFz served as the ground electrode during recording. Participants were asked to avoid blinking, to stay still, and to relax their facial muscles. They were required to look at a fixation square shown on the screen at the beginning of each test trial. To control for eye movement artifacts, horizontal and vertical electrooculograms (EOGs) were recorded from electrodes above the right eye and at the outer canthus of the left eye, respectively (Gratton et al., 1983). Off-line EEG analysis was performed according to a standard procedure using Brain Vision Analyzer Version 2.0.1 (Brain Products GmbH). We analyzed the ERP data without offline filtering in accordance with previous recommendations (Woodman, 2010). The behavioral and EEG data were collected at the same time. The electrode cap was placed on a participant's head during the entirety of an experiment (including the training session and the two UFOV assessments) to optimize the recording of EEG data. The electrode cap was monitored during an experimental session to ensure the impedance for all electrodes was below 5 kΩ.

Raw EEG data were segmented according to the two tasks. All channels were recorded with frontal vertex (i.e., FCz) as the reference and re-referenced to “infinity” zero provided by the reference electrode standardization technique (REST) off-line (Yao, 2001). EEG data were filtered with an IIR bandpass filter between 0.01 and 30 Hz, and were corrected for EOG artifacts using ocular correction. For the analysis, the time epoch for each event was 1,000 ms (200 ms pre-stimulus and 800 ms after the appearance of the stimulus). To avoid eye movement and other artifacts (e.g., blinks, muscle activity, etc.), all epochs exceeding 90 mV in any channel were excluded from additional analysis. For each epoch, a baseline correction for the period 200 ms before stimulus onset was performed. ERPs for experimental conditions were obtained by averaging over trials.

For the power spectrum analysis, after the raw EEG data were pre-processed, three 10 s segments were selected for each participant within each task. A Fast Fourier Transform was computed to generate the power spectrum. Then, the three sections of EEG data for each group were grand averaged. The power spectrum values of the four frequency bands δ (0.5–3.5Hz), θ (3.6–7.5 Hz), α (7.6–12.5 Hz), and β (12.5–25 Hz) were calculated (Bahn et al., 2002). Finally, the relative power theta/alpha value was calculated to reduce the individual differences (Fell et al., 2011). The power spectral density of the signal of the i-th segment is calculated by the periodic graph method. The calculation is shown in Equation (1.1).

(1.1) $$\begin{array}{r} Psd_{i}\left(f\right)=\frac{1}{MU}{\left|\overset{M-1}{\underset{n=0}{\sum }}X_{i}\left(n\right)w\left(n\right)e^{-j2\pi fn}\right|}^{2} \end{array}$$

In the case, i = 1,…, L means the number of segments of signal x(n), j is an imaginary unit, w(n) is a Hamming window function used to reduce the spectral leakage. U is the regularization coefficient of the window, used to reduce the window function on the power spectrum estimation, the calculation is shown in Equation (1.2).


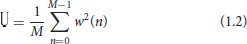


Finally, the power spectral density estimated by the Welch algorithm is the average of each PSD. The calculation is shown in Equation (1.3).

(1.3) $$\begin{array}{r} \text{Psd}\left(f\right)=\frac{1}{L}\overset{L-1}{\underset{i=0}{\sum }}\text{Ps}{\text{d}}_{i}\left(f\right) \end{array}$$

## Results

For each dependent variable, a 2 (group: expert vs. non-experts) × 2 (phase: pre-AVG vs. post-AVG) repeated measures ANOVA was conducted. If a significant main effect or interaction emerged, *post-hoc* analyses were conducted through (*i*) paired-sample *t*-tests that compared the participants' pre- and post-AVG performances and (*ii*) independent samples *t*-tests that analyzed the between-group differences. Adjusted *p*-values were used to correct for multiple comparisons.

### Behavioral data

#### Response time

**Figure 2:**
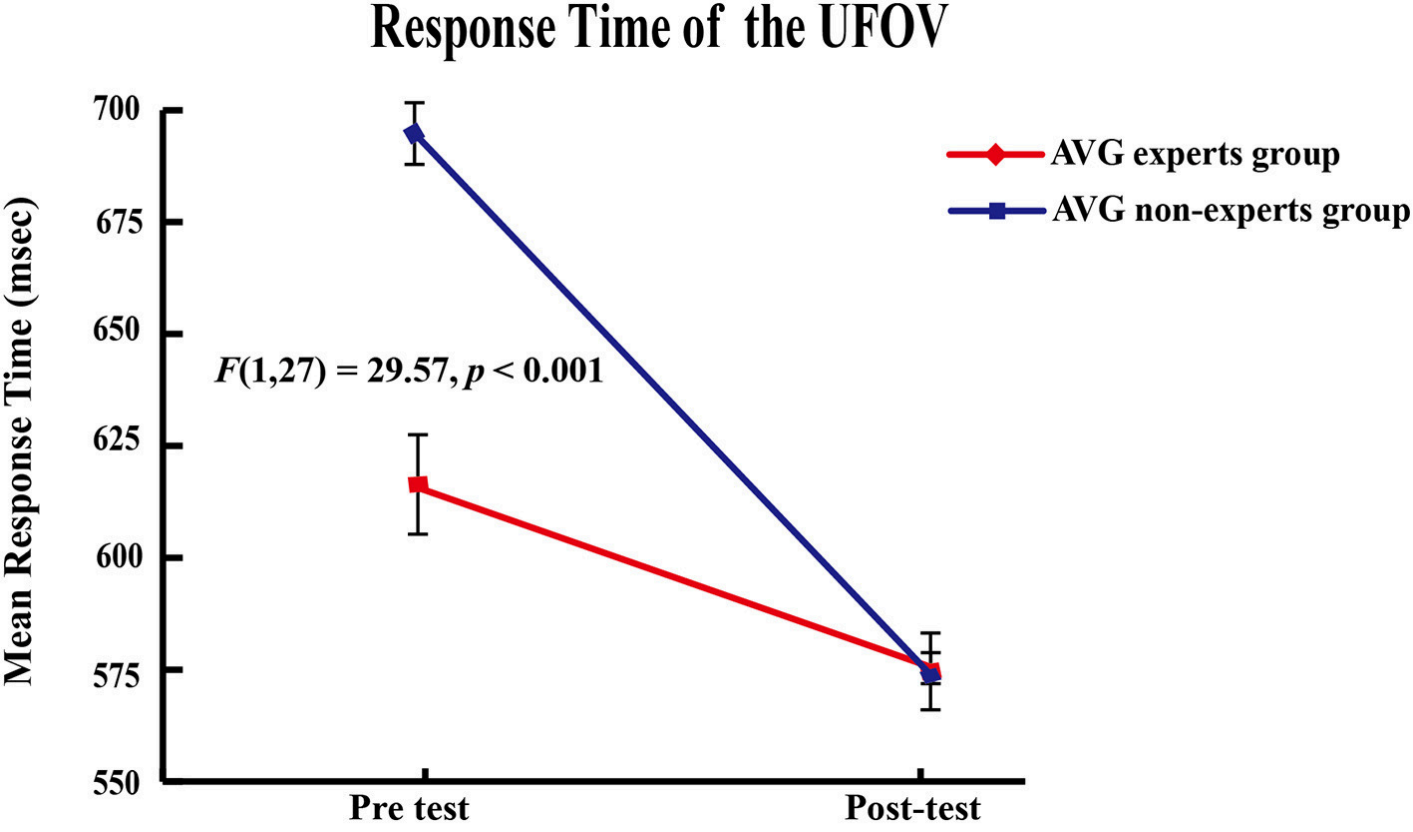
Behavioral results. Significant group × phase interaction effect in response time.

The main effects of group [*F*_(1, 27)_ = 52.53, *p* < 0.001] and phase [*F*_(1, 27)_ = 118.72, *p* < 0.001] and the group × phase interaction [*F*_(1, 27)_ = 29.57, *p* < 0.001] were significant. Paired-sample *t*-tests showed that response time decreased across phases in the experts [*M*_pre_ = 615.30 ms ± 34.77, *M*_post_ = 574.71 ms ± 8.61; *t*_(14)_ = 4.06, *p* < 0.001] and the non-experts [*M*_pre_ = 693.90 ms ± 19.99, *M*_post_ = 572.38 ms ± 26.76; *t*_(13)_ = 10.99, *p* < 0.001]. Independent samples *t* tests showed that the experts had shorter response times than the non-experts [*t*_(27)_ = 7.39, *p* < 0.001] in the pre-AVG phase, but response time did not differ between groups (*p* = 0.75) in the post-AVG phase, suggesting that the non-experts' response times reached the experts' levels after the AVG session (Figure 2).

#### Accuracy

A 2 (group) × 2 (phase) repeated measures ANOVA revealed no significant main effect of group [*F*_(1, 27)_ = 0.12, *p* = 0.73], or a significant group × phase interaction [*F*_(1, 27)_ = 0.63, *p* = 0.43]. Only the main effect of phase [*F*_(1, 27)_ = 16.31, *p* < 0.000] was significant, suggesting that accuracy decreased across phases in both groups. These findings demonstrated that the AVG session did not improve the participants' accuracy in either the experts (*M*_pre_ = 88.64% ± 2.88; *M*_post_ = 84.97% ± 3.56) or the non-experts (*M*_pre_ = 87.68% ± 2.46; *M*_post_ = 85.22% ± 4.68).

### ERP data

**Figure 3:**
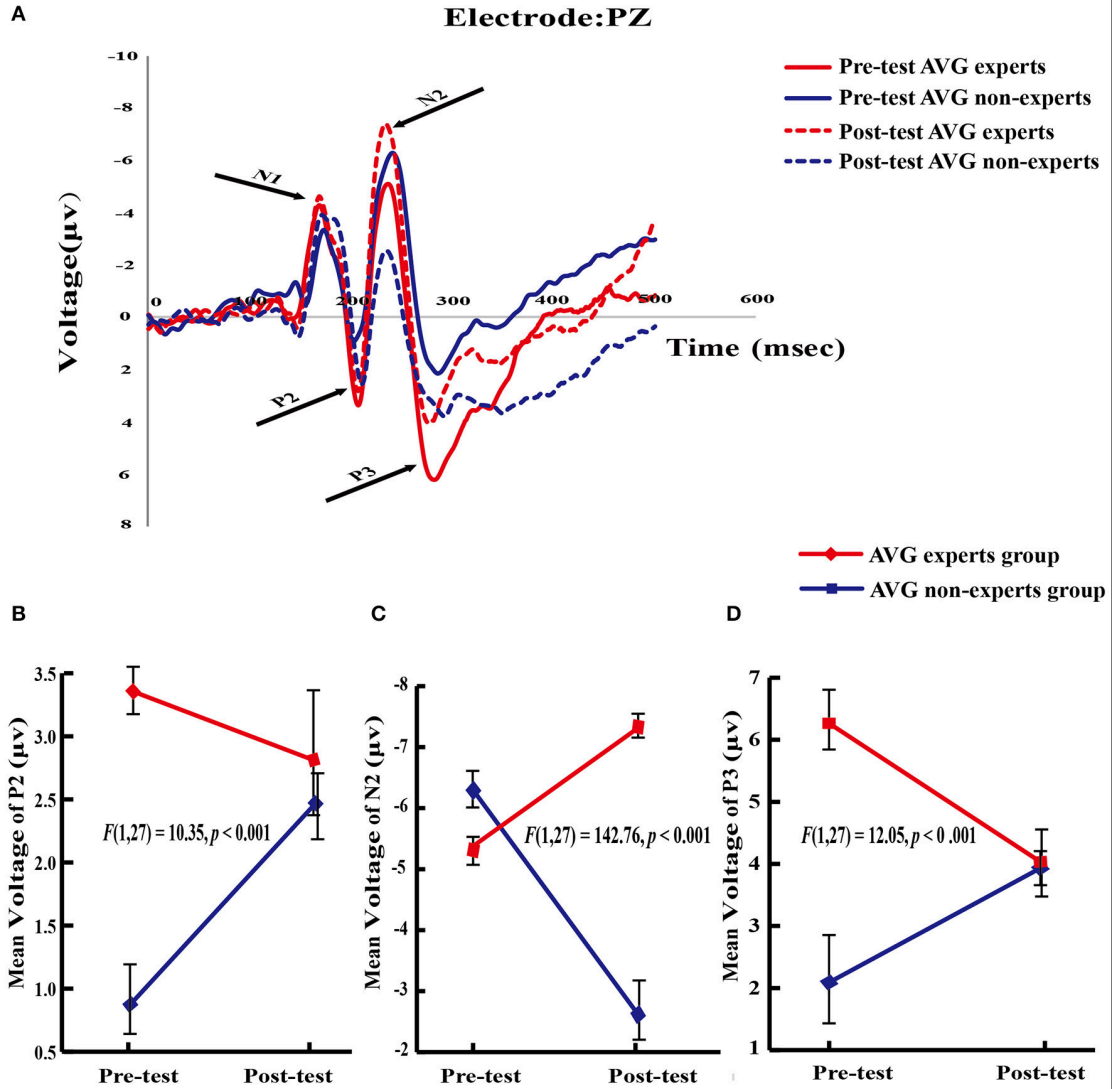
ERPs results. **(A)** Average evoked potentials from theAVG experts group and the non-experts group. N l, P2, N2, and P3 components are shown. **(B–D)** Significant group × phase interaction effect in P2, N2, and P3 potentials.

Based on the literature (Falkenstein et al., 2003; Yao, 2003; Fan et al., 2015), this study analyzed the *amplitudes* of N1, P2, N2, and P3 components (Figure 3A).

#### N1

A 2 (group) × 2 (phase) repeated measures ANOVA revealed no significant main effects or interactions, suggesting that the AVG session used in this study did not change N1 amplitude.

#### P2

The main effect of group [*F*_(1, 27)_ = 8.96, *p* = 0.006] and the group × phase interaction [*F*_(1, 27)_ = 10.35, *p* < 0.001] were significant. Planned paired-sample *t*-tests showed that in the experts, the P2 amplitude did not differ between phases (*M*_pre_ = 3.36 ± 0.91, *M*_post_ = 2.81 ± 2.52; *p* = 0.34). However, in the amateurs, the P2 amplitude increased across phases [*M*_pre_ = 0.87 ± 1.04, *M*_post_ = 2.47 ± 1.09; *t*_(13)_ = 4.85, *p* < 0.001], demonstrating the effect of the training program on the P2 amplitude. Independent samples *t*-tests then analyzed the between-group differences within each phase. The experts had a greater P2 amplitude than the amateurs [*t*_(27)_ = 6.86, *p* < 0.001] in the pre-training phase, revealing the effect of long-term AVG training on the P2 amplitude. However, the P2 amplitude did not differ between groups (*p* = 0.65) in the post-training phase (Figure 3B).

#### N2

The main effects of group [*F*_(1, 27)_ = 20.72, *p* < 0.001] and phase [*F*_(1, 27)_ = 13.73, *p* < 0.001] and the group × phase interaction [*F*_(1, 27)_ = 142.76, *p* < 0.001] were significant. Planned paired-sample *t*-tests showed that N2 amplitude increased across phases in the experts [*M*_pre_ = −5.38 ± 0.93, *M*_post_ = −7.32 ± 0.83; *t*_(14)_ = 8.16, *p* < 0.001] but it decreased across phases in the non-experts [*M*_pre_ = −6.29 ± 1.22, *M*_post_ = −2.62 ± 1.92; *t*_(13)_ = 8.86, *p* < 0.001]. Independent samples *t*-tests showed that compared with the experts, the non-experts had a greater N2 amplitude [*t*_(27)_ = 2.25, *p* = 0.02] in the pre-AVG phase but a smaller N2 amplitude [*t*_(27)_ = 8.67, *p* < 0.001] in the post-AVG phase (Figure 3C).

#### P3

The main effect of group [*F*_(1, 27)_ = 12.74, *p* < 0.001] and the interaction between group and phase [*F*_(1, 27)_ = 12.05, *p* < 0.001] were significant. Planned paired-sample *t*-tests showed that P3 amplitude decreased across phases in the experts [*M*_pre_ = 6.26 ± 1.89, *M*_post_ = 4.03 ± 2.00; *t*_(14)_ = 3.63, *p* < 0.001] but it did not differ between phases in the non-experts (*M*_pre_ = 2.09 ± 3.39; *M*_post_ = 3.93 ± 1.26; *p* = 0.10). Independent samples *t*-tests revealed that the experts had a greater P3 amplitude than the non-experts [*t*_(27)_ = 4.15, *p* < 0.001] in the pre-AVG phase, revealing the association between long-term AVG experience and P3 amplitude. However, P3 amplitude did not differ between groups [*t*_(27)_ = 0.16, *p* = 0.87] in the post-AVG phase (Figure 3D).

#### Power spectrum

**Figure 4:**
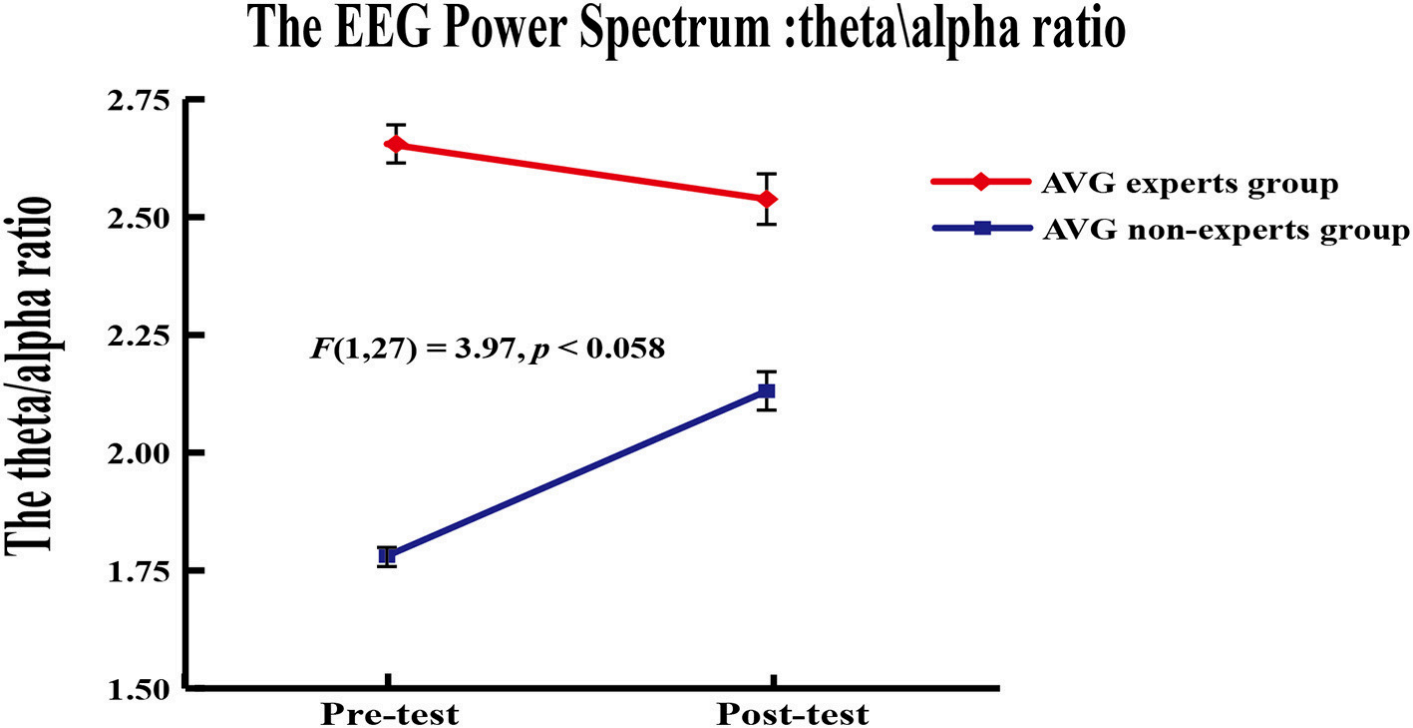
Power spectrum results. The theta/alpha ratio ofEEG power from the AVG experts group and the non-experts group showed a marginally significant interaction in channel Fz.

A 2 (group) × 2 (phase) repeated measures ANOVA was applied to the theta/alpha ratio of EEG power in channel Fz. Results revealed a marginally significant group × phase interaction [*F*_(1, 24)_ = 3.97, *p* = 0.058]. Paired-sample *t*-tests were used to compare the theta/alpha ratio across phases within each group. Results showed that the theta/alpha ratio did not differ between phases in the experts (*M*_pre_ = 2.66 ± 0.41, *M*_post_ = 2.54 ± 0.63; *p* = 0.10) but it increased across phases in the non-experts [*M*_pre_ = 1.78 ± 0.26, *M*_post_ = 2.13 ± 0.45; *t*_(12)_ = 3.04, *p* = 0.01]. Independent samples *t* tests showed that the experts had a greater theta/alpha ratio than the non-experts [*t*_(27)_ = 6.46, *p* < 0.001] in the pre-AVG phase, but the theta/alpha ratio did not differ between groups (*p* = 0.07) in the post-AVG phase (Figure 4).

## Discussion

This study examined whether the plasticity of VSA was observable after only a 1 h AVG session. Both AVG experts and non-experts were recruited and asked to complete VSA assessments prior to and after a 1 h AVG session. This study used behavioral and electrophysiological measures, the latter of which were used because of their high temporal resolution (Carlino et al., 2012). The within-subject analyses compared a participant's performance before and after the AVG session. Results revealed (a) improvements in response time in the experts and non-experts and (b) neural plasticity in the non-experts as indicated by the amplitudes of certain EEG components. Thus, the findings suggested that AVG experience was associated with rapid improvement in VSA. Furthermore, the between-group comparisons showed the relationship between long-term AVG experience and VSA.

### Were the results driven by factors other than AVG?

Was the plasticity of VSA observed in this study driven by a general training effect (or a practice effect) rather than the AVG experience? This is highly unlikely. First, a general training effect tends to improve multiple measures of cognitive performance (Donovan and Radosevich, 1999; Cepeda et al., 2006). However, this study showed that the accuracy was not enhanced after the AVG session. In addition, the observed plasticity (e.g., response time, P2 amplitudes) was associated with indicators highly relevant to VSA, arguing against the possibility that the finding was due to a general training effect. Finally, a general training effect tends to influence all participants. However, the association between a brief AVG experience and neural plasticity was observed only in the non-experts.

Was the plasticity of VSA driven by a transient effect of physiological arousal? First, it should be noted that attention-related EEG measures (e.g., N2, P3) are sensitive indicators to arousal and they tend to change in the same trajectory according to arousal (Rozenkrants et al., 2008; Benikos et al., 2013; Cui et al., 2015). However, we found that *a)* N2 amplitude *increased* across phases in the experts but *decreased* across phases in the non-experts, and *b)* P3 amplitude decreased across phases in the experts but did not differ between phases in the non-experts, suggesting that the current findings were not merely driven by arousal. Second, this study recruited non-experts who had some AVG experience to minimize the influence of novel experience on non-experts' arousal level. Finally, there is evidence suggesting that the effects of video games cannot be simply due to arousal, particularly in experts and highly trained individuals (Jocoy, 2010). Research showed that cognitive performance might be more closely related to the difficulty level of a task (Yerkes and Dodson, 1908; Benikos et al., 2013) than participants' arousal (Barry et al., 2005; VaezMousavi et al., 2009). However, it is possible that the experts had a higher arousal threshold than the non-experts. Although this study does not allow us to rule out this possibility, it should be noted that arousal is an integral part of AVG that likely contributes to the AVG-related learning.

Finally, was the improvement in VSA due to the fact that different UFOV assessments were used prior to and after the AVG session? In other words, perhaps the UFOV assessment used in the post-AVG phase was easier than the one used in the pre-AVG phase. This is also highly unlikely, as accuracy did not increase across phases in either group of participants. Moreover, the purpose of using different UFOV assessments was to eliminate the test-retest practice effect.

### Was the non-experts' VSA enhanced after the 1 h AVG session?

After the AVG session, the non-experts exhibited an increased P2 amplitude, which is indicative of improvements in attentional selection and attentional control processes, including the evaluation of task relevance of the stimulus and the initiation of decision-making (Carretié and Iglesias, 1995; Fritzsche et al., 2011). Furthermore, unlike a previous study using a 10 h AVG program (Wu et al., 2012), the current findings in the non-experts did not show an increased P3 amplitude, which is associated with a top-down modulated increase in the allocation of attentional resources (Polich, 2007). Additionally, we did not find modulations of the N1 component, which is thought to be related to the perception of external physical properties (Grau et al., 2007). Thus, the current study suggests that, after a 1 h AVG session, non-experts may have (a) improvements in cognitive abilities that are closely related to VSA, such as evaluating the task relevance of stimulus and initiating the decision-making process; and (b) modulations of certain EEG components that are highly relevant to VSA.

### How do the effects of long-term and brief AVG experience on VSA interact?

The between-group comparisons showed that the experts had greater P2 and P3 amplitudes than the non-experts prior to the AVG session. Research found that P2 amplitude is related to attentional selection and attentional control processes (Cajochen et al., 1995; Fritzsche et al., 2011), and that P3 amplitude reflects the allocation of attentional resources (Polich, 2007). Thus, the experts' superior VSA may be associated with their long-term AVG experience. However, such a causal inference is hardly conclusive since AVG experts and non-experts may differ in VSA as a result of factors other than AVG experience.

Between-group comparisons showed that P2 and P3 amplitudes did not differ between groups after the AVG session. Thus, the non-experts' VSA could reach expert level after a very brief AVG session. However, these findings by no means suggest that expertise acquisition can be completed within 1 h. Indeed, research has shown that expertise acquisition is a protracted process that may take years to complete (Gong et al., 2013, 2015; Yang et al., 2014). Furthermore, this study also showed that the 1 h AVG training session was related to changes of *only* certain components of VSA. In addition, the retention of the improvements in VSA observed in the non-experts is still unclear.

We also found that N2 amplitude decreased across phases and P2 amplitude increased across phases in the non-experts. Thus, the improvement in VSA in the non-experts might be realized through a re-allocation of cognitive resources—an allocation prioritized for attentional selection and attentional control processes and a reduced resource allocation for the inhibition of irrelevant information. This reallocation of cognitive resources indicates a mechanism that may be important in the early stage of expertise acquisition.

This study also analyzed the relative power spectrum values of theta and alpha. The relative power spectrum values (theta/alpha ratio) may reflect the arousal level (Cajochen et al., 1995; Ma et al., 2012; Huang et al., 2014). We found that the experts had greater theta/alpha ratio than the non-experts in the frontal area before the AVG session but that this advantage was reduced after the AVG session. Consistent with this finding, a recent study on experience of playing driving video game showed that enhanced midline frontal theta power and frontal-posterior theta coherence indicated improved cognitive control (Anguera et al., 2013). Furthermore, cognitive control function is essential for VSA (Lavie et al., 2004). Our results show that both long-term and brief AVG experience may be associated with arousal level, as indexed by theta/alpha ratio.

However, it should be noted that the current experimental design did not allow us to evaluate the retention of the improvements in VSA. Thus, there is a possibility that the improvements observed in this study were a merely transient effect, which differs from learning that is often thought to be associated with stable changes of cognitive performance. Future research should examine whether the improved VSA is maintained after a certain period of washout time (e.g., a full day). Issues related to the time of testing (immediately after play vs. after a wash out period) are important considerations not only for VSA, but also in other fields where investigators seek to examine the impact of video gaming on socio-emotional behavior. For example, an ongoing question in the field of aggression is understanding what can be inferred about long-term changes in behavior from studies that only examine short-term changes. Furthermore, these findings by no means suggest that AVG facilitates human development in all domains, as video gaming experience may (a) increase aggressive behavior, depressive symptoms, and attention deficit symptoms, and (b) reduce pro-social behavior and academic performance (Anderson et al., 2010; Ferguson, 2015). Future research is needed to further explore the effects of AVG on human development.

Furthermore, in the UFOV task, we presented a masking stimulus immediately after the target stimulus to eliminate visual afterimages of the target. Thus, ERPs generated by the mask temporally overlapped the ERPs from the target stimulus, raising the possibility that the mask responses influenced our results. We think this is unlikely, given that the same mask stimuli were used for both groups of participants in this study. Thus, any between-group differences cannot be attributed to effects of the masking stimulus.

## Conclusion

In summary, using both behavioral and electrophysiological measurements, this study revealed that improvement in VSA is observable after a 1 h AVG training session. Furthermore, both long-term and brief AVG experience influenced cognitive processes related to attentional selection and control processes.

## Ethics statement

The experimental protocols were approved by the ethics research committee of the University of Electronic Science and Technology of China (UESTC), and were performed in accordance with ethical standards outlined by the Declaration of Helsinki. Informed consents were obtained from all subjects.

## Author contributions

NQ, DG, and DY contributed to the research design, acquisition, analysis, and interpretation of data, and the writing of this manuscript. WM contributed to the research design, analysis and interpretation of data, and the writing of this manuscript. XF, YZ, YL, YY, ZZ, and FL contributed to analysis and interpretation of data, and the writing of this manuscript. All authors agree to be accountable for all aspects of the work.

### Conflict of interest statement

The authors declare that the research was conducted in the absence of any commercial or financial relationships that could be construed as a potential conflict of interest.

## Footnotes

^1^The LOL consists of three game modes: Summoner's Rift (Ranked Matchmaking), Twisted Treeline, and Howling Abyss (Normal Matchmaking). Players' gaming experience and expertise are indicated by their Ranked Matchmaking level which is calculated based on the Elo rating system—a method for calculating the relative skill levels of players in competitor-versus-competitor games. The experts and non-experts can be defined based on the Elo rating of their Ranked Matchmaking level, which is available from an online inventory (http://www.lol91.com/duanweibilv.html). The Elo rating system has multiple stages—Bronze, Sliver, Gold, Platinum, Diamond, Master, and Challenger—across which the expertise level increases sequentially. Each stage has five phases ranging from V to I. In the Ranked Matchmaking mode, players who win (or lose) a game will gain (or lose) a certain amount of points depending on the champion's performance. Wining each 100 points activates the promotion competition, the successful completion of which promotes the player to the next stage or level. The successful maintenance of points requires one to play LOL on a regular and frequent basis.

## References

[B1] Ackerman P. L. Kanfer R. Calderwood C. (2010). Use it or lose it? Wii brain exercise practice and reading for domain knowledge. Psychol. Aging 25, 753–766. 10.1037/a0019277 20822257 PMC3000863

[B2] Anderson C. A. Shibuya A. Ihori N. Swing E. L. Bushman B. J. Sakamoto A.. (2010). Violent video game effects on aggression, empathy, and prosocial behavior in eastern and western countries: a meta-analytic review. Psychol. Bull. 136, 151–173. 10.1037/a0018251 20192553

[B3] Anguera J. A. Boccanfuso J. Rintoul J. L. Al-Hashimi O. Faraji F. Janowich J.. (2013). Video game training enhances cognitive control in older adults. Nature 501, 97–101. 10.1038/nature12486 24005416 PMC3983066

[B4] Appelbaum L. G. Cain M. S. Darling E. F. Mitroff S. R. (2013). Action video game playing is associated with improved visual sensitivity, but not alterations in visual sensory memory. Attent. Percept. Psychophys. 75, 1161–1167. 10.3758/s13414-013-0472-7 23709062

[B5] Bahn E. Nolte W. Kurth C. Ramadori G. Rüther E. Wiltfang J. (2002). Quantification of the electroencephalographic theta/alpha ratio for the assessment of portal-systemic encephalopathy following implantation of transjugular intrahepatic portosystemic stent shunt (TIPSS). Metab. Brain Dis. 17, 19–28. 10.1023/A:1014048229754 11893005

[B6] Ball K. Wadley V. Edwards J. (2002). Advances in technology used to assess and retrain older drivers. Gerontechnology 1, 251–261. 10.4017/gt.2002.01.04.004.00

[B7] Barry R. J. Clarke A. R. McCarthy R. Selikowitz M. Rushby J. A. (2005). Arousal and activation in a continuous performance task - an exploration of state effects in normal children. J. Psychophysiol. 19, 91–99. 10.1027/0269-8803.19.2.91

[B8] Bavelier D. Achtman R. L. Mani M. Föcker J. (2012). Neural bases of selective attention in action video game players. Vis. Res. 61, 132–143. 10.1016/j.visres.2011.08.007 21864560 PMC3260403

[B9] Bejjanki V. R. Zhang R. Li R. Pouget A. Green C. S. Lu Z. L.. (2014). Action video game play facilitates the development of better perceptual templates. Proc. Natl. Acad. Sci. U.S.A. 111, 16961–16966. 10.1073/pnas.1417056111 25385590 PMC4250112

[B10] Benikos N. Johnstone S. J. Roodenrys S. J. (2013). Varying task difficulty in the Go/Nogo task: the effects of inhibitory control, arousal, and perceived effort on ERP components. Int. J. Psychophysiol. 87, 262–272. 10.1016/j.ijpsycho.2012.08.005 22902315

[B11] Blacker K. Curby K. M. Klobusicky E. Chein J. M. (2014). Effects of action video game training on visual working memory. J. Exp. Psychol. Hum. Percept. Perform. 40, 1992–2004. 10.1037/a0037556 25068696

[B12] Blacker K. J. Curby K. M. (2013). Enhanced visual short-term memory in action video game players. Attent. Percept. Psychophys. 75, 1128–1136. 10.3758/s13414-013-0487-0 23709068

[B13] Boduroglu A. Shah P. (2017). Cultural differences in attentional breadth and resolution. Cult. Brain 5, 169–181. 10.1007/s40167-017-0056-9

[B14] Boot W. R. Champion M. Blakely D. P. Wright T. Souders D. J. Charness N. (2013). Video games as a means to reduce age-related cognitive decline: attitudes, compliance, and effectiveness. Front. Psychol. 4:31. 10.3389/fpsyg.2013.00031 23378841 PMC3561600

[B15] Cajochen C. Brunner D. P. Kräuchi K. Graw P. Wirz-Justice A. (1995). Power density in theta/alpha frequencies of the waking EEG progressively increases during sustained wakefulness. Sleep 18, 890–894. 10.1093/sleep/18.10.890 8746397

[B16] Cardoso-Leite P. Bavelier D. (2014). Video game play, attention, and learning: how to shape the development of attention and influence learning? Curr. Opin. Neurol. 27, 185–191. 10.1097/wco.0000000000000077 24553464

[B17] Carlino E. Sigaudo M. Pollo A. Benedetti F. Mongini T. Castagna F.. (2012). Nonlinear analysis of electroencephalogram at rest and during cognitive tasks in patients with schizophrenia. J. Psychiatry Neurosci. 37, 259–266. 10.1503/jpn.110030 22353633 PMC3380097

[B18] Carretié L. Iglesias J. (1995). An ERP study on the specificity of facial expression processing. Int. J. Psychophysiol. 19, 183–192. 10.1016/0167-8760(95)00004-C 7558985

[B19] Cepeda N. J. Pashler H. Vul E. Wixted J. T. Rohrer D. (2006). Distributed practice in verbal recall tasks: a review and quantitative synthesis. Psychol. Bull. 132, 354–380. 10.1037/0033-2909.132.3.354 16719566

[B20] Colzato L. S. van den Wildenberg W. P. Zmigrod S. Hommel B. (2012). Action video gaming and cognitive control: playing first person shooter games is associated with improvement in working memory but not action inhibition. Psychol. Res. 77, 1237–1239. 10.1007/s00426-012-0415-2 22270615

[B21] Connors E. C. Chrastil E. R. Sánchez J. Merabet L. B. (2014). Action video game play and transfer of navigation and spatial cognition skills in adolescents who are blind. Front. Hum. Neurosci. 8:133. 10.3389/fnhum.2014.00133 24653690 PMC3949101

[B22] Cui F. Zhu X. Duan F. Luo Y. (2015). Instructions of cooperation and competition influence the neural responses to others' pain: an ERP study. Soc. Neurosci. 11, 289–296. 10.1080/17470919.2015.1078258 26226618

[B23] Dale G. Green C. S. (2017a). Associations between avid action and real-time strategy game play and cognitive performance: a pilot study. J. Cogn. Enhance. 1, 295–317. 10.1007/s41465-017-0021-8

[B24] Dale G. Green C. S. (2017b). The changing face of video games and video gamers: future directions in the scientific study of video game play and cognitive performance. J. Cogn. Enhance. 1, 280–294. 10.1007/s41465-017-0015-6

[B25] Donchin E. Coles M. G. H. (1988). Is the P300 component a manifestation of context updating? Behav. Brain Sci. 11, 357–374. 10.1017/s0140525x00058027

[B26] Donovan J. Radosevich D. (1999). A meta-analytic review of the distribution of practice effect: now you see it, now you don't. J. Appl. Psychol. 84, 795–805. 10.1037/0021-9010.84.5.795

[B27] Drollette E. S. Scudder M. R. Raine L. B. Moore R. D. Saliba B. J. Pontifex M. B.. (2014). Acute exercise facilitates brain function and cognition in children who need it most: an ERP study of individual differences in inhibitory control capacity. Dev. Cogn. Neurosci. 7, 53–64. 10.1016/j.dcn.2013.11.001 24309300 PMC6987893

[B28] Dye M. W. Green C. S. Bavelier D. (2009). The development of attention skills in action video game players. Neuropsychologia 47, 1780–1789. 10.1016/j.neuropsychologia.2009.02.002 19428410 PMC2680769

[B29] Falkenstein M. Hoormann J. Hohnsbein J. Kleinsorge T. (2003). Short-term mobilization of processing resources is revealed in the event-related potential. Psychophysiology 40, 914–923. 10.1111/1469-8986.00109 14986844

[B30] Fan Y. Tang Y. Y. Tang R. Posner M. I. (2015). Time course of conflict processing modulated by brief meditation training. Front. Psychol. 6:911. 10.3389/fpsyg.2015.00911 26191022 PMC4490222

[B31] Fell J. Ludowig E. Staresina B. P. Wagner T. Kranz T. Elger C. E.. (2011). Medial temporal theta/alpha power enhancement precedes successful memory encoding: evidence based on intracranial EEG. J. Neurosci 31, 5392–5397. 10.1523/jneurosci.3668-10.2011 21471374 PMC6622710

[B32] Feng J. Spence I. (2007). Effects of cognitive training on individual differences in attention, in Engineering Psychology and Cognitive Ergonomics: 7th International Conference, EPCE 2007, Held as Part of HCI International 2007, Beijing, China, July 22-27, 2007, Proceedings, ed Harris D. (Berlin; Heidelberg: Springer), 279–287.

[B33] Feng J. Spence I. Pratt J. (2007). Playing an action video game reduces gender differences in spatial cognition. Psychol. Sci. 18, 850–855. 10.1111/j.1467-9280.2007.01990.x 17894600

[B34] Ferguson C. J. (2015). Do angry birds make for angry children? A meta-analysis of video game influences on children's and adolescents' aggression, mental health, prosocial behavior, and academic performance. Perspecti. Psychol. Sci. 10, 646–666. 10.1177/1745691615592234 26386002

[B35] Fritzsche A. S. Stahl J. Gibbons H. (2011). An ERP study of target competition: individual differences in functional impulsive behavior. Int. J. Psychophysiol. 81, 12–21. 10.1016/j.ijpsycho.2011.03.014 21510982

[B36] Glass B. D. Maddox W. T. Love B. C. (2013). Real-time strategy game training: emergence of a cognitive flexibility trait. PLoS ONE 8:e70350. 10.1371/journal.pone.0070350 23950921 PMC3737212

[B37] Gong D. He H. Liu D. Ma W. Dong L. Luo C.. (2015). Enhanced functional connectivity and increased gray matter volume of insula related to action video game playing. Sci. Rep. 5:9763. 10.1038/srep09763 25880157 PMC5381748

[B38] Gong D. He H. Ma W. Liu D. Huang M. Dong L.. (2016). Functional integration between salience and central executive networks: a role for action video game experience. Neural Plasticity 2016:9803165. 10.1155/2016/9803165 26885408 PMC4739029

[B39] Gong D. Ma W. Kendrick K. M. Hu Q. Yao D. (2013). How cognitive plasticity resolves the brain's information processing dilemma. Sci. Rep. 3:2860. 10.1038/srep02860 24091591 PMC3790200

[B40] Gratton G. Coles M. G. Donchin E. (1983). A new method for off-line removal of ocular artifact. Electroencephalogr. Clin. Neurophysiol. 55, 468–484. 10.1016/0013-4694(83)90135-9 6187540

[B41] Grau C. Fuentemilla L. Marco-Pallarés J. (2007). Functional neural dynamics underlying auditory event-related N1 and N1 suppression response. Neuroimage 36, 522–531. 10.1016/j.neuroimage.2007.03.027 17499521

[B42] Green C. S. Bavelier D. (2003). Action video game modifies visual selective attention. Nature 423, 534–537. 10.1038/nature01647 12774121

[B43] Green C. S. Bavelier D. (2006). Effect of action video games on the spatial distribution of visuospatial attention. J. Exp. Psychol. Hum. Percept. Perform. 32, 1465–1478. 10.1037/0096-1523.32.6.1465 17154785 PMC2896828

[B44] Green C. S. Bavelier D. (2015). Action video game training for cognitive enhancement. Curr. Opin. Behav. Sci. 4, 103–108. 10.1016/j.cobeha.2015.04.012

[B45] Green C. S. Li R. Bavelier D. (2010). Perceptual learning during action video game playing. Top. Cogn. Sci. 2, 202–216. 10.1111/j.1756-8765.2009.01054.x 25163784

[B46] Gross C. G. Rocha-Miranda C. E. Bender D. B. (1972). Visual properties of neurons in inferotemporal cortex of the Macaque. J. Neurophysiol. 35, 96–111. 10.1152/jn.1972.35.1.96 4621506

[B47] Huang S. Rossi S. Hämäläinen M. Ahveninen J. (2014). Auditory conflict resolution correlates with medial–lateral frontal theta/alpha phase synchrony. PLoS ONE 9:e110989. 10.1371/journal.pone.0110989 25343503 PMC4208834

[B48] Hultsch D. F. Hertzog C. Small B. J. Dixon R. A. (1999). Use it or lose it: engaged lifestyle as a buffer of cognitive decline in aging? Psychol. Aging 14, 245–263. 10403712 10.1037//0882-7974.14.2.245

[B49] Jasper H. H. (1958). The ten twenty electrode system of the international federation. Electroencephalogr. Clin. Neurophysiol. 10, 371–375. 10590970

[B50] Jocoy K. A. (2010). Stress-Induced Analgesia Through Video Game Play. Dissertation/master's thesis, Appalachian State University.

[B51] Krishnan L. Kang A. Sperling G. Srinivasan R. (2013). Neural strategies for selective attention distinguish fast-action video game players. Brain Topogr. 26, 83–97. 10.1007/s10548-012-0232-3 22614909 PMC3536985

[B52] Kundu B. Sutterer D. W. Emrich S. M. Postle B. R. (2013). Strengthened effective connectivity underlies transfer of working memory training to tests of short-term memory and attention. J. Neurosci. 33, 8705–8715. 10.1523/JNEUROSCI.5565-12.2013 23678114 PMC3758887

[B53] Latham A. J. Patston L. L. Tippett L. J. (2013). The virtual brain: 30 years of video-game play and cognitive abilities. Front. Psychol. 4:629. 10.3389/fpsyg.2013.00629 24062712 PMC3772618

[B54] Lavie N. Hirst A. de Fockert J. W. Viding E. (2004). Load theory of selective attention and cognitive control. J. Exp. Psychol. Gen. 133, 339–354. 10.1037/0096-3445.133.3.339 15355143

[B55] Li R. Polat U. Makous W. Bavelier D. (2009). Enhancing the contrast sensitivity function through action video game training. Nat. Neurosci. 12:549. 10.1038/nn.2296 19330003 PMC2921999

[B56] Lövdén M. Bäckman L. Lindenberger U. Schaefer S. Schmiedek F. (2010). A theoretical framework for the study of adult cognitive plasticity. Psychol. Bull. 136, 659–676. 10.1037/a0020080 20565172

[B57] Ma W. Lai Y. Yuan Y. Wu D. Yao D. (2012). Electroencephalogram variations in the α band during tempo-specific perception. Neuroreport 23, 125–128. 10.1097/WNR.0b013e32834e7eac 22186801

[B58] Milne E. Dunn S. A. Freeth M. Rosas-Martinez L. (2013). Visual search performance is predicted by the degree to which selective attention to features modulates the ERP between 350 and 600 ms. Neuropsychologia 51, 1109–1118. 10.1016/j.neuropsychologia.2013.03.002 23499721

[B59] Oei A. C. Patterson M. D. (2014). Are videogame training gains specific or general? Front. Syst. Neurosci. 8:54 10.3389/fnsys.2014.00054 24782722 PMC3986546

[B60] Oldfield R. C. (1971). The assessment and analysis of handedness: the Edinburgh inventory. Neuropsychologia 9, 97–113. 10.1016/0028-3932(71)90067-4 5146491

[B61] Owen A. M. Hampshire A. Grahn J. A. Stenton R. Dajani S. Burns A. S.. (2010). Putting brain training to the test. Nature 465, 775–778. 10.1038/nature09042 20407435 PMC2884087

[B62] Polich J. (2007). Updating P300: an integrative theory of P3a and P3b. Clini. Neurophysiol. 118, 2128–2148. 10.1016/j.clinph.2007.04.019 17573239 PMC2715154

[B63] Powers K. L. Brooks P. J. Aldrich N. J. Palladino M. A. Alfieri L. (2013). Effects of video-game play on information processing: a meta-analytic investigation. Psychon. Bull. Rev. 20, 1055–1079. 10.3758/s13423-013-0418-z 23519430

[B64] Rozenkrants B. Olofsson J. K. Polich J. (2008). Affective visual event-related potentials: arousal, valence, and repetition effects for normal and distorted pictures. Int. J. Psychophysiol. 67, 114–123. 10.1016/j.ijpsycho.2007.10.010 18160161 PMC2674071

[B65] Salthouse T. A. (2006). Mental exercise and mental aging:evaluating the validity of the “use it or lose it” hypothesis. Perspect. Psychol. Sci. 1, 68–87. 10.1111/j.1745-6916.2006.00005.x 26151186

[B66] Schubert T. Finke K. Redel P. Kluckow S. Müller H. Strobach T. (2015). Video game experience and its influence on visual attention parameters: an investigation using the framework of the Theory of Visual Attention (TVA). Acta Psychol. 157, 200–214. 10.1016/j.actpsy.2015.03.005 25834984

[B67] Sekuler A. B. Bennett P. J. Mamelak M. (2000). Effects of aging on the useful field of view. Exp. Aging Res. 26, 103–120. 10.1080/036107300243588 10755218

[B68] Serences J. T. Yantis S. Culberson A. Awh E. (2004). Preparatory activity in visual cortex indexes distractor suppression during covert spatial orienting. J. Neurophysiol. 92, 3538–3545. 10.1152/jn.00435.2004 15254075

[B69] Shors T. J. Anderson M. L. Curlik D. M. Nokia M. S. (2012). Use it or lose it: how neurogenesis keeps the brain fit for learning. Behav. Brain Res. 227, 450–458. 10.1016/j.bbr.2011.04.023 21536076 PMC3191246

[B70] Sungur H. Boduroglu A. (2012). Action video game players form more detailed representation of objects. Acta Psychol. 139, 327–334. 10.1016/j.actpsy.2011.12.002 22266223

[B71] Thomas C. Baker C. I. (2013). Teaching an adult brain new tricks: a critical review of evidence for training-dependent structural plasticity in humans. Neuroimage 73, 225–236. 10.1016/j.neuroimage.2012.03.069 22484409

[B72] Toril P. (2014). Video game training enhances cognition of older adults: a meta-analytic study. Psychol. Aging 29, 706–717. 10.1037/a0037507 25244488

[B73] VaezMousavi S. M. Barry R. J. Clarke A. R. (2009). Individual differences in task-related activation and performance. Physiol. Behav. 98, 326–330. 10.1016/j.physbeh.2009.06.007 19545581

[B74] Wang P. Liu H. H. Zhu X. T. Meng T. Li H. J. Zuo X. N. (2016). Action video game training for healthy adults: a meta-analytic study. Front. Psychol. 7:907. 10.3389/fpsyg.2016.00907 27378996 PMC4911405

[B75] West G. L. Al-Aidroos N. Pratt J. (2013). Action video game experience affects oculomotor performance. Acta Psychol. 142, 38–42. 10.1016/j.actpsy.2011.08.005 23220058

[B76] West G. L. Drisdelle B. L. Konishi K. Jackson J. Jolicoeur P. Bohbot V. D. (2015). Habitual action video game playing is associated with caudate nucleus-dependent navigational strategies. Proceedings. Biol. Sci. 282:20142952. 10.1098/rspb.2014.2952 25994669 PMC4455792

[B77] West G. L. Stevens S. A. Pun C. Pratt J. (2008). Visuospatial experience modulates attentional capture: evidence from action video game players. J. Vis. 8:13. 10.1167/8.16.13 19146279

[B78] Woodman G. (2010). A brief introduction to the use of event-related potentials in studies of perception and attention. Attent. Percept. Psychophys. 72, 2031–2046. 10.3758/BF03196680 21097848 PMC3816929

[B79] Wu S. Cheng C. K. Feng J. D'Angelo L. Alain C. Spence I. (2012). Playing a first-person shooter video game induces neuroplastic change. J. Cogn. Neurosci. 24, 1286–1293. 10.1162/jocn_a_00192 22264193

[B80] Wu S. Spence I. (2013). Playing shooter and driving videogames improves top-down guidance in visual search. Attent. Percept. Psychophys. 75, 673–686. 10.3758/s13414-013-0440-2 23460295

[B81] Yang H. Ma W. Gong D. Hu J. Yao D. (2014). A longitudinal study on children's music training experience and academic development. Sci. Rep. 4:5854. 10.1038/srep05854 25068398 PMC5376161

[B82] Yao D. (2001). A method to standardize a reference of scalp EEG recordings to a point at infinity. Physiol. Meas. 22, 693–711. 10.1088/0967-3334/22/4/305 11761077

[B83] Yao D. (2003). Computation for the implicit components of ERP in attention. Brain Topogr. 16, 65–70. 10.1023/A:1025662500837 14587970

[B84] Yerkes R. M. Dodson J. D. (1908). The relation of strength of stimulus to rapidity of habit-formation. J. Comp. Neurol. Psychol. 18, 459–482. 10.1002/cne.920180503

